# PD-L1 Activity Is Associated with Partial EMT and Metabolic Reprogramming in Carcinomas

**DOI:** 10.3390/curroncol29110654

**Published:** 2022-10-31

**Authors:** Srinath Muralidharan, Manas Sehgal, R. Soundharya, Susmita Mandal, Sauma Suvra Majumdar, M. Yeshwanth, Aryamaan Saha, Mohit Kumar Jolly

**Affiliations:** 1 Centre for BioSystems Science and Engineering, Indian Institute of Science, Bangalore 560012, India; slakshmivenkates@mdanderson.org (S.M.); manassehgal@iisc.ac.in (M.S.); srbt20112@student.nitw.ac.in (R.S.); susmitam@iisc.ac.in (S.M.); yeshwanthm@iisc.ac.in (M.Y.); 2 Department of Biotechnology, National Institute of Technology, Durgapur 713216, India; ssm18u10669@btech.nitdgp.ac.in; 3 Department of Biotechnology, Indian Institute of Technology Madras, Chennai 600036, India; be19b014@smail.iitm.ac.in

**Keywords:** partial EMT, meta-analysis, immune checkpoint molecules, glycolysis, oxidative phosphorylation, metabolic plasticity

## Abstract

Immune evasion and metabolic reprogramming are hallmarks of cancer progression often associated with a poor prognosis and frequently present significant challenges for cancer therapies. Recent studies have highlighted the dynamic interaction between immunosuppression and the dysregulation of energy metabolism in modulating the tumor microenvironment to promote cancer aggressiveness. However, a pan-cancer association among these two hallmarks, and a potent common driver for them—epithelial-mesenchymal transition (EMT)—remains to be done. This meta-analysis across 184 publicly available transcriptomic datasets as well as The Cancer Genome Atlas (TCGA) data reveals that an enhanced PD-L1 activity signature along with other immune checkpoint markers correlate positively with a partial EMT and an elevated glycolysis signature but a reduced OXPHOS signature in many carcinomas. These trends were also recapitulated in single-cell, RNA-seq, time-course EMT induction data across cell lines. Furthermore, across multiple cancer types, concurrent enrichment of glycolysis and PD-L1 results in worse outcomes in terms of overall survival as compared to enrichment for only PD-L1 activity or expression. These results highlight potential functional synergy among these interconnected axes of cellular plasticity in enabling metastasis and multi-drug resistance in cancer.

## 1. Introduction

The dynamic interplay between host immunity and cancer cells often facilitates tumor development and is now recognized as a cancer hallmark [1,2]. Cancer cells exploit a myriad of counter-regulatory mechanisms, which are crucial for self-tolerance, limiting the host immune attack and creating a tumor-promoting, immune-suppressive milieu [3,4]. Many immune checkpoint molecules such as the cluster of differentiation 274 (CD274) that encodes for programmed death receptor-ligand 1 (PD-L1) protein [5], cytotoxic T-lymphocyte–associated antigen 4 (CTLA4) [6], cluster of differentiation 276 (CD276) [7], lymphocyte-activation gene 3 (LAG3) [8], and hepatitis A virus cellular receptor 2 (HAVCR2) [9] are known to block anti-tumor responses in the tumor microenvironment [3]. Particularly, the programmed death receptor-ligand 1 (PD-L1) plays a crucial role in the metastatic spread of tumors by binding to programmed death receptor 1 (PD-1) on CD8+ T cells, thus imparting a potent immune evasive character to cancer cells mainly by altering effector functions of T cells, along with the inhibition of T cell proliferation and survival [10,11]. Accumulating evidence suggests that cancer cells can exhibit high PD-L1 expression [12,13,14] and this upregulation can be influenced by multiple signaling pathways [10,15]. Like PD-1, CTLA4 is an inhibitory receptor that prevents CD28-mediated co-stimulatory signals for immune response [6], while LAG3 contributes to a state of T cell dysfunction in the tumor microenvironment [8]. Additionally, CD276 and HAVC2 are emerging as new immune checkpoint genes that participate in immune infiltration and thereby serve as clinical target to improve the therapeutic efficacy of immunotherapy [9,16]. Thus, an understanding of the diverse array of regulatory mechanisms that govern immune evasion and their implications for therapeutic intervention in cancer is an active area of research. 

In this regard, the upregulation of PD-L1-mediated immunosuppression by cancer cells has been extensively associated with epithelial-mesenchymal transition (EMT), wherein epithelial cells which possess apicobasal polarity and are bound by cell-cell junctions lose their characteristic epithelial markers while gaining mesenchymal features like spindle shape and enhanced motility. The process of EMT also allows for cells to achieve hybrid epithelial/mesenchymal (E/M) states through a multi-step transition. EMT is often implicated in cancer metastasis and therapy resistance, making it crucial for aggressive cancer progression [17,18,19,20,21]. Moreover, cells with this hybrid E/M phenotype can express elevated levels of PD-L1 [18,22]. On a different note, emerging evidence supports the notion that cells undergoing EMT also exhibit varying degrees of metabolic reprogramming in pathological conditions such as cancer [23]. Alterations in key metabolic pathways such as glycolysis, oxidative phosphorylation, lipid metabolism, and amino acid metabolism influence cancer progression at least partly by modulating the EMT status of cells [24,25]. More recently, metabolic reprogramming has been increasingly linked with immune evasion [1,26,27]. For instance, increased glycolytic metabolism of cancer cells favors cancer growth by competing with T cells for growth and blocking lactic acid export in T cells, thus impeding the cytotoxic activity of T cells [27,28]. Concomitantly, inhibition of PD-L1 expression, when coupled with a blockade of lactate production enhances anti-tumor effects of metformin by boosting T-cell function and limiting cancer cell proliferation [29]. Further, modulation of oxidative phosphorylation (OXPHOS) for overcoming PD-1 resistance to improve anti-tumor responses in specific cancers has been observed [27,30]. These findings suggest that the reprogrammed metabolic axes and enrichment of immune evasion markers can complement each other in driving cancer progression [27,29,31,32,33,34]. However, a detailed pan-cancer analysis of such coupling has not yet been conducted.

Here, through a meta-analysis of 184 transcriptomic datasets (Table S1A), primary tumor samples in multiple carcinomas in The Cancer Genome Atlas (TCGA), and single-cell RNA sequencing data, we evaluated the relationship between these metabolic reprogramming axes and PD-L1 activity and analyzed consequences of this association for disease prognosis. We observed a predominant positive correlation of an elevated PD-L1 signature (and CD274 expression levels) with enrichment of partial EMT and glycolysis signatures, but negative correlation with an OXPHOS signatures. Such trends were also largely consistent in a single-cell, time-course EMT induction dataset, as well as with other immune checkpoint molecules. Finally, these results revealed that concurrent enrichment of PD-L1 and glycolysis associate with worse patient survival than just glycolysis enrichment without PD-L1, thereby indicating possible synergy in functional aspects of immune suppression and metabolic plasticity in cancer progression. 

## 2. Methods

### 2.1. Software and Datasets

The computational and statistical analyses were conducted using R (version 4.2.1) and Python (version 3.9). Microarray and single-cell RNA sequencing datasets were retrieved from NCBI GEO (Gene expression omnibus) using the ‘GEOquery’ R package. RNA sequencing FASTQ files were obtained from the ENA (European Nucleotide Archive) database. TCGA datasets were obtained using UCSC Xena tools. 

### 2.2. Pre-Processing of Datasets

The pre-processing of the microarray datasets was conducted to obtain the gene-wise expression from the probe-wise expression matrix using respective annotation files for the mapping of probes to genes. In case multiple probes mapped to a single gene, the mean expression of all mapped probes was utilized to obtain the final values for those genes.

The overall quality of the RNA sequencing datasets was assessed using FastQC. Adapter trimming of FASTQ files was done using ‘Trimmomatic’ (version 0.39) [35] and STAR aligner (version 2.7.10a) [36] was used for the alignment of the reads with hg38-human (or mm10-mouse) reference genome. Raw counts were calculated using HTseq-count and were then normalized for gene length and transformed to TPM (transcripts per million) values. They were then log2 normalized to acquire the final expression data.

For single-cell RNA sequencing datasets, the MAGIC (version 2.0.3) [37] imputation algorithm was utilized to recover noisy and sparse single-cell data using diffusion geometry. Relevant platform annotation files were utilized to map individual reads to particular genes.

### 2.3. EMT Scoring Methods

For each dataset, several methods were employed to determine EMT scores. Each technique necessitates the input of gene expression data along with a different gene set algorithm.

#### 2.3.1. 76GS and KS Scores

76GS scores for each sample were calculated using a gene set containing 76 genes [38]. Each sample’s weighted total of the gene expression levels of the 76 genes was calculated, with correlation coefficients to CDH1 expression levels serving as the weighting factors. According to this new scale, a low 76GS score represents a more mesenchymal phenotype, whereas a high value indicates the predominance of an epithelial one. 

The KS technique scores EMT for cell lines and tumor samples using the two-sample Kolmogorov–Smirnov test (KS) [39]. For each of the two signatures (E and M), cumulative distribution functions (CDFs) are produced, and the largest difference between these CDFs is utilized as the test statistic for a two-sample KS test. The resultant EMT scores are in the range [−1, 1]. Mesenchymal and epithelial phenotypes are indicated by positive and negative scores, respectively.

#### 2.3.2. Epithelial and Mesenchymal Scores

ssGSEA (single sample gene set enrichment analysis) was performed on KS epithelial (for Epi scores) and KS mesenchymal (for Mes scores) gene lists separately using the GSEAPY python library to quantify the enrichment of epithelial and mesenchymal signatures independently. The enrichment scores indicate the degree of cordial up/down-regulation of genes in the gene set for a given sample. The normalized enrichment score (NES) for these gene sets was obtained for further analysis. A higher Epi score denotes a more epithelial phenotype, whereas a higher Mes score signifies the enrichment of a mesenchymal phenotype.

#### 2.3.3. Hallmark EMT and Partial EMT Scores

The ssGSEA method available in the GSEAPY python was used to calculate NES for hallmark EMT geneset from the molecular signatures database (MSigDB) [40]. Partial EMT (pEMT) geneset [41] was utilized to calculate NES for pEMT scores.

### 2.4. Scoring Methods for Metabolic Pathways and PD-L1

ssGSEA scores were calculated for the hallmark pathway genesets from MSigDB (see supplementary Table S1B) to obtain the respective normalized gene signature enrichment scores.

AMPK and HIF-1α signatures were quantified using expression levels of their downstream target genes, as previously reported [42]. In total, 33 downstream genes for AMPK and 23 downstream genes for HIF-1α were used. FAO2 scores were calculated based on equations previously reported, which use the expression levels of 14 enzyme genes related to FAO [43]. PD-L1 signature was curated as reported earlier [22], wherein the top correlated genes (Spearman correlation coefficient > 0.5 and *p* < 0.01) with PD-L1 levels in at least any 15 out of the 27 cancer types were considered for this analysis.

The activity scores for metabolic and E/M signatures for the single-cell RNA sequencing datasets were computed using AUCell (version 1.18.1) [44] from the R package ‘Bioconductor’ with default parameters.

### 2.5. Survival Analysis

Survival data (overall survival) were acquired from TCGA. Based on the median of the sample scores, all samples were split into two groups: high PD-L1 and high glycolysis (’P+G+’) and high PD-L1 but low glycolysis (’P+G−’). The R package ’survival’ was employed to perform the Kaplan–Meier analysis, and the plotting was done using ‘ggfortify’. Reported *p*-values were calculated using a log-rank test. Cox regression was used to determine the hazard ratio (HR) and confidence interval (95% CI) for TCGA cohorts, and forest plots were made using ‘ggplot2’.

## 3. Results 

### 3.1. Enrichment of PD-L1 Signature Is Associated with Partial-EMT

To assess the association of PD-L1 with the EMT status of cells, we first calculated single-sample gene set enrichment analysis (ssGSEA) scores for each sample in 184 bulk transcriptomic datasets (Table S1A) for PD-L1, KS epithelial (Epi), KS mesenchymal (Mes) and partial-EMT gene lists (Table S1B,C). We also calculated 76GS and KS scores for individual samples in these datasets (Table S1C). Higher Epi and 76GS scores signify the enrichment of an epithelial program, whereas a higher KS or Mes score indicates the prevalence of a mesenchymal one.

**Figure 1:**
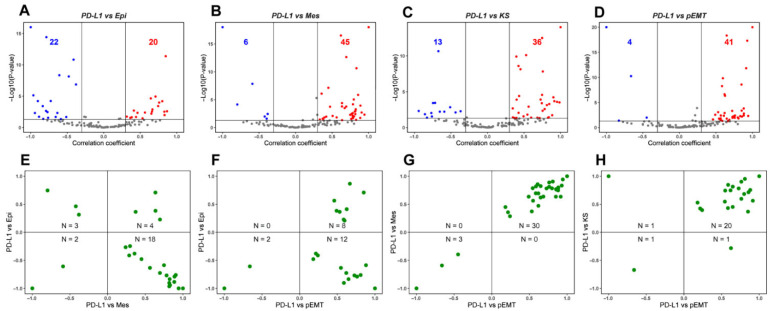
Association between PD-L1 activity gene signature and partial-EMT. (**A**) Volcano plots showing the correlation coefficient, R (x-axis), and -log_10_ (*p*-value) (y-axis) for PD-L1 vs. Epi scores. Boundaries for significant correlation are set at |R| > 0.3 and *p* < 0.05. Same as (**A**) but for (**B**) PD-L1 vs. Mes, (**C**) PD-L1 vs. KS and (**D**) PD-L1 vs. pEMT scores. (**E**) Two-dimensional scatter plot depicting the correlation coefficient ‘R’ between PD-L1 vs. Mes (x-axis) and PD-L1 vs. Epi scores (y-axis). ‘N’ denotes the number of significant data points lying in each quadrant. Same as (**E**) but for (**F**) PD-L1 vs. pEMT and PD-L1 vs. Epi, (**G**) PD-L1 vs. pEMT and PD-L1 vs. Mes and (**H**) PD-L1 vs. pEMT and PD-L1 vs. KS scores. Each dot denotes a dataset for which this correlation is calculated.

On correlating the PD-L1 activity signature [22] with Epi scores, a comparable number of datasets showed the correlation to be either positive (*n* = 20; red data points) and negative (*n* = 22; blue data points) (Figure 1A). On the other hand, PD-L1 gene set activity showed a consistent positive correlation with both Mes and KS scores. Out of 51 datasets that showed a significant correlation for the PD-L1 vs. Mes scores, 45 of them (88.23%) showed a positive correlation (Figure 1B). Similarly, 73.46% (36 out of 49) datasets showed a positive correlation between KS scores and PD-L1 activity (Figure 1C). Further, PD-L1 scores showed a strong positive skew (41 out of 45 = 91.11%) with the activity of partial EMT geneset [41] (Figure 1D). Together, these results together suggest a link between an enriched PD-L1 signature with a partial EMT program, wherein the cancer cells may acquire a hybrid E/M phenotype rather than being completely epithelial or mesenchymal in nature. 

Next, we plotted the correlation coefficients of these pairwise comparisons to further investigate the putative link between PD-L1 and EMT spectrum. In this analysis, we only considered datasets that were significant across both pairwise comparisons. 64.28% (18 out of 28) datasets showed a positive link between PD-L1 and Mes scores while being negatively associated with Epi scores (Figure 1E). In 91.30% (20 out of 22) datasets, PD-L1 activity correlated positively with pEMT scores, among which the correlation with Epi scores can be in either direction to a comparable extent (Figure 1F). Further, among the 33 datasets in which PD-L1 activity was associated significantly both with Mes and KS scores, 30 datasets (91.17%) had a positive correlation of PD-L1 activity with both the KS and Mes scores (Figure 1G). Reinforcing trends were seen for the association of PD-L1 activity with pEMT signature one (Figure 1H). Thus, this pan-cancer meta-analysis highlights that the prominent mode of association in these pairwise comparisons is that PD-L1 activity correlates positively with a partial mesenchymal nature, reminiscent of recent experimental reports [18,22,45]. 

To characterize the connection of immune evasion and EMT extensively, we examined the association of additional immune checkpoint genes with an EMT program. Gene-wise expression values of immune checkpoints: CD274, CD276, CD47, CTLA4, HAVCR2, LAG3, LGALS9 (encodes Galectin-9 protein), and PDCD1 (encodes programmed cell death 1 receptor) were correlated with 76GS, KS, and pEMT scores. CD274, CD276, and CD47 correlated predominantly positively with 76GS, KS and pEMT scores (Figure S1A–C), indicating their probable link with partial EMT. Although HAVCR2 and LGALS9 also showed similar trends with pEMT scores, their correlation with 76GS and KS scores did not show a strong skew (Figure S1E,G). Further, CTLA4, PDCD1, and LAG3 had no strong trends in terms of correlation with 76GS, KS, or pEMT scores (Figure S1D,F,H). Overall, most immune checkpoint genes are associated positively with partial EMT, similar to the trends seen for the PD-L1 gene signature.

Together, across this cohort of datasets spanning multiple cancer types (Table S1A), while an enriched PD-L1 gene signature (and gene expression of many immune checkpoints analyzed) did not associate strongly with an epithelial program in either direction (positive or negative), they showed a predominant positive correlation with a mesenchymal signature. This trend was strengthened by the strong positive association of PD-L1 and immune checkpoint genes with the pEMT gene set. Therefore, these results consistently indicate the association of immune evasion with partial EMT in many carcinomas. 

### 3.2. PD-L1 Enrichment Is Linked to an Upregulated Glycolysis Signature

**Figure 2:**
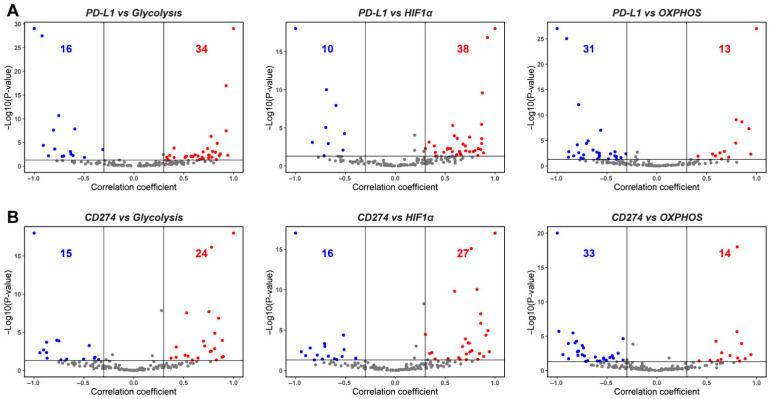

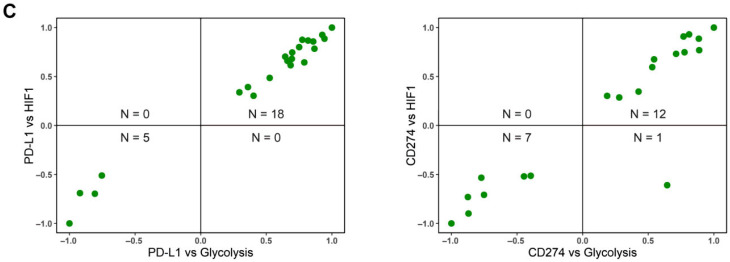
Association between PD-L1, glycolysis, and OXPHOS. (**A**) Volcano plots showing the correlation coefficient, ‘R’ (x-axis), and -log_10_ (*p*-value) (y-axis) for PD-L1 vs. glycolysis scores (**left**) and PD-L1 vs. HIF-1α (**middle**) and PD-L1 vs. OXPHOS (**right**). Boundaries for significant correlation are set at R > ± 0.3 and *p* < 0.05. Same as (**A**) but for (**B**), CD274 gene expression vs. glycolysis (**left**)**,** CD274 gene expression vs. HIF-1α (**middle**), and CD274 gene expression vs. OXPHOS (**right**). (**C**) Two-dimensional scatter plot depicting correlation coefficient ‘R’ between PD-L1 vs. glycolysis (x-axis) and PD-L1 vs. HIF-1α scores (y-axis) (**left**) and CD274 expression vs. glycolysis (x-axis) and CD274 vs. HIF-1α scores (y-axis) (**right**). ‘N’ denotes the number of significant data points lying in each quadrant.

After examining the association of PD-L1 activity with EMT, we assessed its association with major aspects of energy metabolism that are known to undergo variable degrees of reprogramming during tumor progression, notably Glycolysis and OXPHOS. In the context of cancer progression, several independent studies link the upregulation of a glycolytic program and downregulation of OXPHOS with the enrichment of PD-L1 [26,31,34]. Consistent with these observations, we observed a strong positive correlation of PD-L1 activity with a glycolysis-associated gene set and with Hypoxia-inducible factor 1 alpha (HIF-1α), a key glycolytic player. Out of 50 datasets that displayed a significant correlation for the PD-L1 and glycolysis scores, 68% (*n* = 34) datasets show a positive association. PD-L1 activity scores correlate even more strongly with the HIF-1α signature, where 38 out of 48 cases (79.17%) reflect a positive correlation between the two. Conversely, among 44 datasets where PD-L1 scores correlated with OXPHOS activity scores, 31 of them (70.45%) exhibited a negative association between the two (Figure 2A, Table S2). These trends were recapitulated in the correlation of expression levels of CD274 with the glycolysis, HIF-1α, and OXPHOS signatures (Figure 2B). Upon considering pairwise comparisons across individual datasets, glycolysis and HIF-1α enrichment are consistently associated with upregulated PD-L1 signature and CD274 expression (Figure 2C).

Among the additional immune checkpoint markers considered here, expression levels of most of them—CD276, CTLA4, HAVCR2, LAG3, and PDCD1—correlated negatively with OXPHOS. CD47 and CD276 also predominantly exhibited a positive association with HIF1α and/or glycolysis scores (Figure S2A,B), while CTLA4, LAG3, and PDCD1 were most likely to be negatively associated with glycolysis (Figure S2C–G). These results suggest that glycolysis and OXPHOS may not always be strongly mutually antagonistic to one another, thereby suggesting the possibility of hybrid metabolic (high glycolysis/high OXPHOS) and metabolically quiescent (low glycolysis/low OXPHOS) states, besides the canonical high glycolysis/low OXPHOS and high OXPHOS/low glycolysis states [43,46].

Overall, both PD-L1 activity scores and CD274 expression levels associate strongly positively with glycolysis and negatively with OXPHOS, consistent with the largely antagonistic trend established for glycolysis and OXPHOS programs in the context of cancer progression [42]. Further, these strong trends indicate a possible cooperative association between metabolic reprogramming and immune suppression in a pan-cancer manner.

### 3.3. Immune Checkpoint Markers Correlate Positively with Partial EMT, PD-L1, and Immune-Response Signatures in Adenocarcinomas

Multiple inflammatory pathways such as tumor necrosis factor-alpha (TNF-α), interferon-gamma (IFN-γ), and nuclear factor-kappa B (NF-κB) are known to induce PD-L1 [47]. Another immune checkpoint maFrker, LGALS9, was reported to be involved in regulating many of these inflammatory pathways [48]. Thus, we conducted a correlation analysis to elucidate the connection between CD274 (PD-L1 gene) and immune checkpoint markers used earlier (CD47, CD276, CTLA4, HAVCR2, LAG3, LGALS9, and PDCD1) (Table S3). We used well-characterized cohorts of primary tumor data from TCGA in a tissue-specific manner: breast cancer (BRCA), prostate adenocarcinoma (PRAD), bladder cancer (BLCA), stomach adenocarcinomas (STAD), lung adenocarcinoma (LUAD), and pancreatic adenocarcinoma (PAAD).

**Figure 3:**
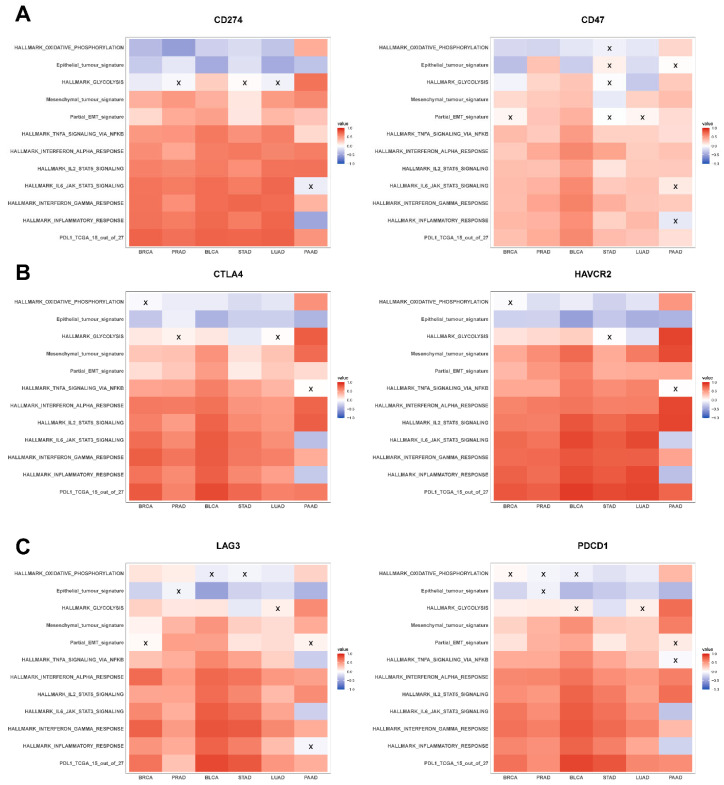
Association of immune checkpoint markers with several hallmark gene signatures in TCGA cohorts: (**A**) heatmap illustrating the Spearman correlation coefficients between different hallmark gene signatures with CD274 (**left**) and CD47 gene expression (**right**) in BRCA, PRAD, BLCA, STAD, LUAD, and PAAD. Insignificant correlations (*p* > 0.05) are marked with ‘X’. Same as (**A**) but for (**B**) CTLA4 (**left**) and HAVCR2 gene expression data (**right**) and (**C**) LAG3 (**left**) and PDCD1 gene expression data (**right**).

Across the six abovementioned carcinomas, except occasional deviations seen in PAAD, CD274 expression levels correlated positively with a mesenchymal and a partial EMT signature but correlated negatively with both the hallmark OXPHOS pathway and epithelial signatures (Figure 3A, left), consistent with the meta-analysis presented earlier (Figure 1 and Figure 2). While the association of CD274 expression levels with glycolysis gene set was not as strong across cancer types, they correlated positively with hallmark pathways associated with immune response and inflammation such as INF-α response, IFN-γ response, TNF-α signaling via NF-κB, and IL-2/STAT5 signaling (Figure 3A, left).

The abovementioned relationships were largely seen also with the other immune checkpoint genes, where CD47, CTLA4, HAVCR2, LAG3, LGALS9, and PDCD1 correlated positively with metabolic reprogramming and inflammatory signatures, as well as with the mesenchymal and partial EMT ones (Figure 3A–C and Figure S3A). However, the negative correlation with epithelial signature was noticed in only four out of eight immune checkpoint genes (Figure 3 and Figure S3A). Additionally, we noticed that all the immune checkpoint genes (except CD276) were strongly positively correlated with the PD-L1 signature while also positively associating with each other (Figure 3 and Figure S3B).

In conclusion, in primary tumor samples in TCGA, multiple immune checkpoint molecules, including CD274, correlated positively with inflammatory and immune response-associated pathways, a PD-L1 signature, and with mesenchymal behavior. However, the association of these molecules with an epithelial behavior was not as consistently and strongly negative across carcinomas, reminiscent of the previous observations of CD274 expression and PD-L1 signature associating with a partial EMT phenotype. These results augment the trends seen in in vitro pan-cancer datasets earlier, establishing a predominant overlap among the enhanced expression of immune checkpoint molecules, partial EMT, and metabolic reprogramming aspects.

### 3.4. Association of CD274 Gene Expression with Partial EMT and Metabolic Reprogramming Is Recapitulated in Single-Cell RNA Sequencing Data

To further probe the different modalities of association between the EMT status and metabolism reprogramming with PD-L1, we conducted a similar analysis in a single-cell RNA sequencing dataset (GSE147405) [49]. From this dataset, we analyzed the transcriptomic profiles of three cell lines (A549, DU145, and OVCA420) treated with two different EMT-inducers (TGF-β, TNF-α) to analyze how CD274 expression levels change alongside alterations in epithelial-mesenchymal and cellular metabolism status (Table S4A). As a first step, we validated that the EMT scoring metrics used here (KS, Epi, Mes) Table were consistent in quantifying the E/M status of cells in this dataset (Figure S4C, Table S4B). While the KS score correlated positively with the Mes scores consistently across cell lines and treatments, their correlation with Epi scores depended on the cell line—it was expectedly negative in OVCA420, but weakly positive in the two cell lines where the Epi and Mes programs were not as strongly antagonistic to one another—A549, DU145. These results further endorse that downregulation of epithelial traits and upregulation of mesenchymal ones may not necessarily happen simultaneously, as often tacitly assumed while claiming EMT [50,51]. 

**Figure 4:**
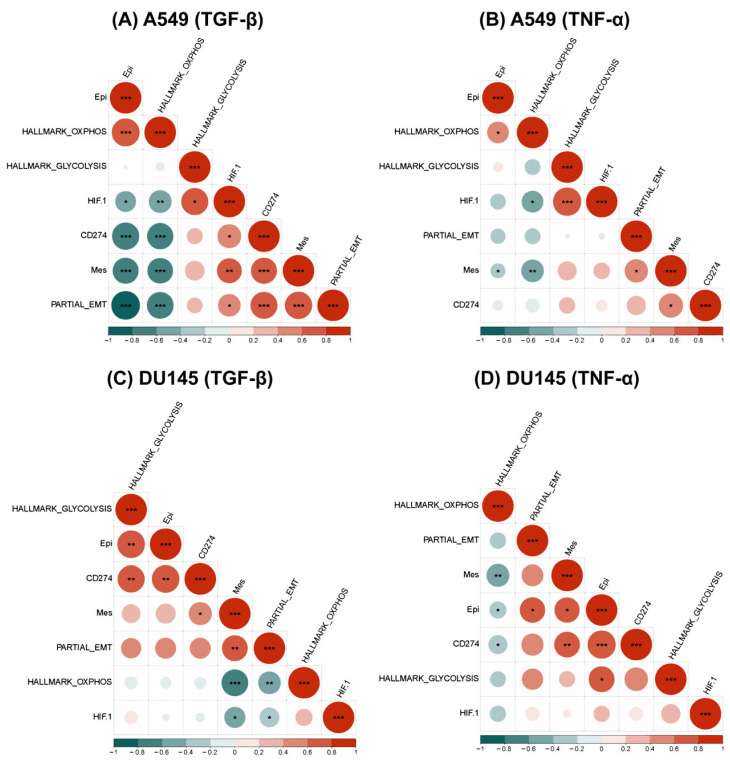
Association between EMT and metabolic axes with CD274 gene expression in single-cell RNA sequencing data (GSE147405). (**A**) Heatmap illustrating correlation coefficient ‘R’ for EMT metrics, metabolic pathways, and the CD274 gene in TGF-β-treated A549 cell line. *p*-values are calculated using unpaired Students’ T-test with unequal variance and significant correlations are marked with an asterisk (*) for *p* < 0.05; **: *p* <0.01; ***: *p* < 0.001. (**B**) Same as (**A**) but for TNF-α-treated A549 cell line. (**C**) TGF-β-treated DU145 cell line and (**D**) TNF-α-treated DU145 cell line.

In this single-cell dataset, we observed that activity of the mesenchymal gene set correlated positively with CD274 expression across both treatments, further validating the trends observed in bulk datasets (Figure 4 and Figure S4). Additionally, CD274 expression leaned strongly towards the positive direction with the epithelial signature in TNF-α-treated DU145 and OVCA420 samples (Figure 4C and Figure S4B) while associating significantly negatively in TGF-β-treated A549 case (Figure 4A). Epi scores were generally non-committed to either direction in the remaining samples (Figure 4B,C and Figure S4A). Apart from the TNF-α-treated DU145 sample, in all other cell lines, CD274 correlated positively with pEMT (Figure 4 and Figure S4A,B). These results support the observations made in bulk datasets and corroborate the association of CD274 with a partial EMT program. 

We also noticed that in A549 and DU145 cell lines treated with TGF-β and TNF-α, the OXPHOS signature consistently displayed a negative correlation with CD274 expression, as seen previously with PD-L1 and OXPHOS in bulk data. Additionally, the glycolysis gene signature revealed a positive correlation between CD274 gene expression levels and PD-L1 gene expression values in bulk data; however, this correlation was statistically significant only in TGF-β-treated DU145 cells (Figure 4C,D). On the contrary, in OVCA420 cells treated with TGF-β, both the hallmark OXPHOS and glycolysis signatures showed a significant negative correlation with CD274 expression (Figure S4A). Although, the negative trend was more pronounced with OXPHOS as compared to glycolysis, these results showcase that glycolysis and OXPHOS are not as mutually antagonistic as often presumed. Furthermore, HIF-1α has a significantly positive correlation with CD274 expression.

Therefore, the association of CD274 gene expression with an epithelial-hybrid-mesenchymal status of cells is reflected in this analysis of this single-cell dataset, which substantiates previous reports [22] and the analysis of bulk transcriptomic datasets. Moreover, similar trends were witnessed for major axes of energy metabolism in cancer cell glycolysis and OXPHOS, with cell line and/or treatment-specific variations altering the extent of correlation seen in this single-cell, time-course data [23]. 

### 3.5. Survival Analysis Reveals the Association of Concomitant Enrichment of PD-L1 and Glycolysis with Worse Patient Survival

**Figure 5:**
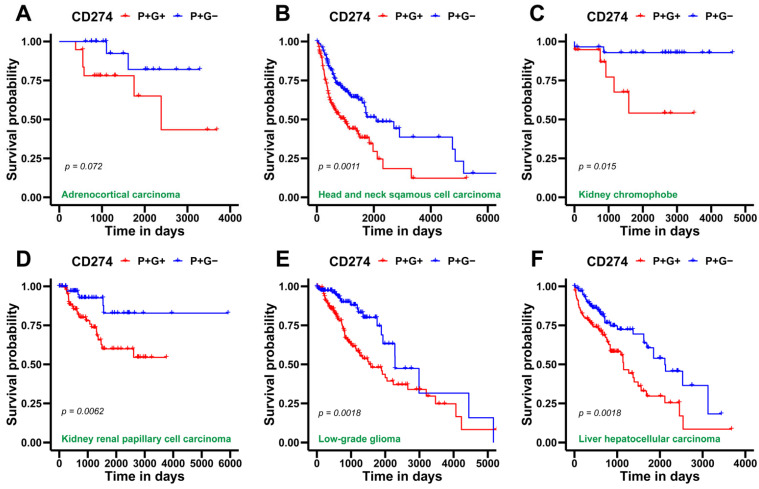

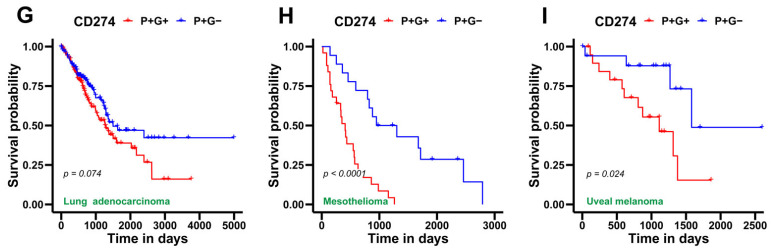
Survival analysis for CD274 gene expression and glycolysis gene signature in several TCGA patient cohorts across cancer types. (**A**) Kaplan–Meier curves associating overall survival (OS) with both a high CD274 gene expression and glycolysis signature (blue) and a high CD274 gene expression but a low glycolysis signature (red) in adenoid cystic carcinoma cohort from TCGA. Reported p-values are based on a log-rank test a indicating significant difference in survival. Same as (**A**) but for (**B**), head and neck squamous cell carcinoma, (**C**) kidney chromophobe, (**D**) kidney renal papillary cell carcinoma, (**E**) low-grade glioma, (**F**) liver hepatocellular carcinoma, (**G**) lung adenocarcinoma, (**H**) mesothelioma, and (**I**) uveal melanoma.

Finally, we obtained CD274 gene expression and glycolysis scores of patient samples from TCGA across various cancer types to identify any association of metabolic reprogramming and/or immune-suppressive aspects with patient survival. For this assessment, we utilized overall survival (OS) data for two segregated sample groups, one with a high CD274 and glycolysis score: P+G+ (blue curve in Figure 5); and the other with a high CD274 expression but low glycolysis score: P+G− (red curve in Figure 5). We observed that P+G+ samples were associated with significantly worse patient survival when compared to the P+G− across multiple cancers, indicating that a concurrent upregulation of CD274 expression and glycolysis signatures results in more aggressive disease progression in a pan-cancer manner (Figure 5). Repeating the same analysis with the PD-L1 signature gene set yielded a similar trend, wherein the P+G− samples corresponded with higher OS probability in all TCGA cohorts considered for this analysis (Figure S5). 

After examining the association of OS data for patient samples with PD-L1 and glycolysis, we investigated the hazard function associated with the expression of immune checkpoint markers previously analyzed in each cancer type in TCGA datasets (Figure S6). Log_2_ hazard ratios (Log_2_HR) corresponding to overall survival for each scenario were calculated (Table S5). Log_2_HR > 0 indicates an increased risk of morbidity, whereas Log_2_HR < 0 signifies better overall survival. This analysis revealed a more context-specific association of the gene expression of immune checkpoint markers with patient outcomes. Significant Log_2_HR values reveal the association of higher CD274 expression with worse survival for skin cutaneous melanoma (SKCM), kidney renal clear-cell carcinoma (KIRC), and adrenocortical carcinoma (ACC), while there is a better patient outcome for low-grade glioma (LGG) (Figure S6A). Elevated CD276 gene expression was predominantly linked with better overall survival (Figure S6B). Log_2_HR ratios were less than zero for CTLA4 gene expression in LGG and KIRC, while being higher than zero in SKCM, head and neck squamous carcinoma (HNSC), and breast invasive carcinoma (BRCA) (Figure S6C). Higher HAVCR2 gene expression was associated with a lower risk in uveal melanoma (UVM), LGG, thymoma (THYM), and esophageal carcinoma (ESCA), but higher in cervical squamous cell carcinoma and endocervical adenocarcinoma (CESC) and SKCM (Figure S6D). Additionally, increased LAG3 expression was associated with better survival outcome in UVM, LGG, and KIRC but worse in SKCM, thyroid carcinoma (THCA), and mesothelioma (MESO) (Figure S6E). For high LGALS9 expression, CESC, BRCA, bladder urothelial carcinoma (BLCA), HNSC, MESO, sarcoma (SARC), and SKCM showed worse prognostic probability, while having better patient outcomes for UVM, LGG, and KIRC (Figure S6F). At last, a higher PDCD1 gene expression was linked to lower Log_2_HR in UVM, LGG, kidney renal papillary cell carcinoma (KIRP), and ESCA, but high Log_2_HR for HNSC, SKCM, uterine corpus endometrial carcinoma (UCEC) and BRCA (Figure S6G).

In all, these pan-cancer observations offer insights on the classification of patient samples with more vs. less PD-L1 (or CD274) and glycolysis, as well as the worse probability of patient survival linked with their parallel enrichment, which is largely uniform across all evaluated cancer types. In contrast, immune checkpoint gene expression displayed a contextual relationship with overall survival probabilities for different cancer types. 

## 4. Discussion

Tumors often dysregulate the expression of key immune checkpoint proteins [3]. One such protein, called programmed death ligand-1 (PD-L1), is often employed by cancer cells to bypass the host immunity. The programmed cell death protein-1 (PD-1) on active cytotoxic-T lymphocytes (CTLs) that have infiltrated tumors is detected by PD-L1 on cancer cells and macrophages that effectively turn off their ‘cancer-clearing’ activity through multiple mechanisms. Moreover, compared to normal tissues, tumor tissues express PD-L1 at considerably higher levels, which drove interest in inhibiting the PD-L1/PD-1 signaling axis as an appealing strategy for cancer immunotherapy [52,53,54]. However, increasing occurrences of resistance in such immunotherapy-based treatments have been reported [55]. It is thus essential to understand the underlying mechanisms behind adaptive immune evasion and its regulation by other molecular factors in the tumor microenvironment (TME) to improve the efficacy of immune checkpoint blockade therapy. In this context, this pan-cancer analysis focuses on specific hallmarks of cancer: EMT and metabolic reprogramming in cancer cells and their association with immune evasion to discern the role of PD-L1 and other checkpoint proteins in cancer progression.

Increasing evidence suggests that PD-L1 regulation is associated with the EMT status of cancer cells. For instance, in many carcinomas—BRCA, ESCA, and non-small cell lung carcinoma (NSCLC)—the EMT status of cells strongly associates with PD-L1 expression levels [56], at least partly through the action of pathways such as phosphoinositide 3-kinase/protein kinase B pathway [57]. Moreover, many EMT-TFs are known modulators of PD-L1 expression, enabling cells in one or more hybrid E/M phenotype(s) to have enriched PD-L1 levels [58,59]. 

Previous work, including ours, has also indicated the association of EMT with metabolic reprogramming [60,61,62]. Thus, the switching of cancer cell energetics from aerobic respiration (or OXPHOS) to an anaerobic one (or glycolysis)—called the Warburg effect—can impact EMT as well as TME to alter immune-evasive traits. While many cancer cells exhibit a strong propensity towards glycolysis to acclimate themselves with the hypoxic conditions in TME, several studies report that oxidative phosphorylation can remain intact in many different cancers and in a context-specific manner, thus enabling hybrid metabolic phenotypes akin to the ones reported for EMT extensively now [46]. Such reprogramming can influence both cancer cell behavior and TME to display immunosuppressive characteristics [30,63,64]. A primary reason for this is that immunosuppression can be competition in TME brought on by increased glucose demand of cancer cells. Consistently, a study reported that specifically targeting PD-L1 with monoclonal antibodies resulted in decreased glycolysis in tumor cells via an obstruction of the PI3K/Akt/mTOR pathway and the translation of glycolytic enzymes, thereby improving the anti-tumor function of T cells [55]. These results reinforce the meta-analysis observations that PD-L1 expression and activity levels are positively correlated with the glycolysis pathway in bulk microarray and RNA sequencing datasets, with cancer-specific differences shaping the trends in TCGA cohort and single-cell analysis. Our observations on the correlation between HIF-1α and PD-L1 resonate with experimental observations in tumor-bearing mice reporting that PD-L1 upregulation in hypoxia depended on HIF-1α activity [65]. 

Although this analysis suggests possible synergy among the different hallmarks of tumor progression, we did not perform any specific analysis to elucidate the direction of the mechanistic influence of these cellular programs precisely. Thus, a causal relationship among these axes needs to be yet elucidated through specific perturbation experiments. Such patterns of association could help in developing more effective anti-tumor strategies in the future to tackle the clinical challenges of tumor plasticity and heterogeneity that tend to improve the fitness of cancer often as a whole [66]. 

## Data Availability

Publicly available transcriptomics datasets from NCBI GEO (Table S1A), TCGA cohorts and single-cell RNA sequencing data from David Cook et al. [49] were analyzed in this study. Codes used to perform the analyses shown in this manuscript are available at https://github.com/Srinath6762/PD-L1-Metabolism (accessed on 1 September 2022).

## Supplementary Materials

The following supporting information can be downloaded at: www.mdpi.com/xxx/s1, Figure S1: Relationship of immune checkpoint markers with EMT in bulk transcriptomics datasets, Figure S2: Relationship of immune checkpoint markers with glycolysis (its master regulator HIF-1α) and OXPHOS gene signatures, Figure S3: Association of immune checkpoint markers with hallmark pathways in TCGA datasets, Figure S4: Association between EMT and metabolic axes with CD274 gene expression in single-cell RNA sequencing data (GSE147405), Figure S5: Survival analysis for PD-L1 and glycolysis gene signatures in several TCGA patient cohorts across cancer types, Figure S6: Cox proportional hazard ratios for immune checkpoint markers in different TCGA datasets, Table S1A: Description of the 184 bulk transcriptomic datasets used in this study, Table S1B: Gene sets used for scoring EMT, metabolic and PD-L1 gene signatures, Table S1C: Spearman’s Correlation coefficient ‘R’ and corresponding *p*-values for correlation of PD-L1 gene signature and immune checkpoint markers with KS, 76GS, Epi, Mes and pEMT scores, Table S2: Spearman’s Correlation coefficient ‘R’ and corresponding *p*-values for correlation of PD-L1 gene signature and immune checkpoint markers with glycolysis, HIF-1α, and OXPHOS scores, Table S3: Spearman’s Correlation coefficient ‘R’ and corresponding *p*-values for correlation of immune checkpoint markers with different hallmark pathways in TCGA cohorts, Table S4A: Spearman’s Correlation coefficient ‘R’ and corresponding *p*-values for correlation between CD274 gene expression, E/M scores, and scores for metabolism in cell line samples of GSE147405, Table S4B: Spearman’s Correlation coefficient ‘R’ and corresponding *p*-values for correlation between KS vs. Mes, Epi vs. Mes, and Ks vs. Epi for cell line samples in GSE147405, Table S5: Log2 hazard ratios, mean ± 95% confidence intervals and corresponding *p*-values for overall survival associated with CD274 gene expression across several TCGA cancer types.
