# Supplementary material for: PD-L1 Activity Is Associated with Partial EMT and Metabolic Reprogramming in Carcinomas
Source: Curr Oncol. 2022 Oct 31;29(11):8285–301. doi: 10.3390/curroncol29110654 (PMC9688938; doi:10.3390/curroncol29110654)
Supplement: Supplementary file 1 [file curroncol-29-00654-s001.zip › curroncol-1982582-supplementary for conversion 10.31.pdf]

Article

PD-L1 Activity is Associated with Partial EMT and Metabolic Reprogramming in Carcinomas

Srinath Muralidharan [1,†], Manas Sehgal [1,†], Soundharya R [1,†], Susmita Mandal [1], Sauma Suvra Majumdar [2], Yeshwanth M [1], Aryamaan Saha [3] and Mohit Kumar Jolly [1,*]

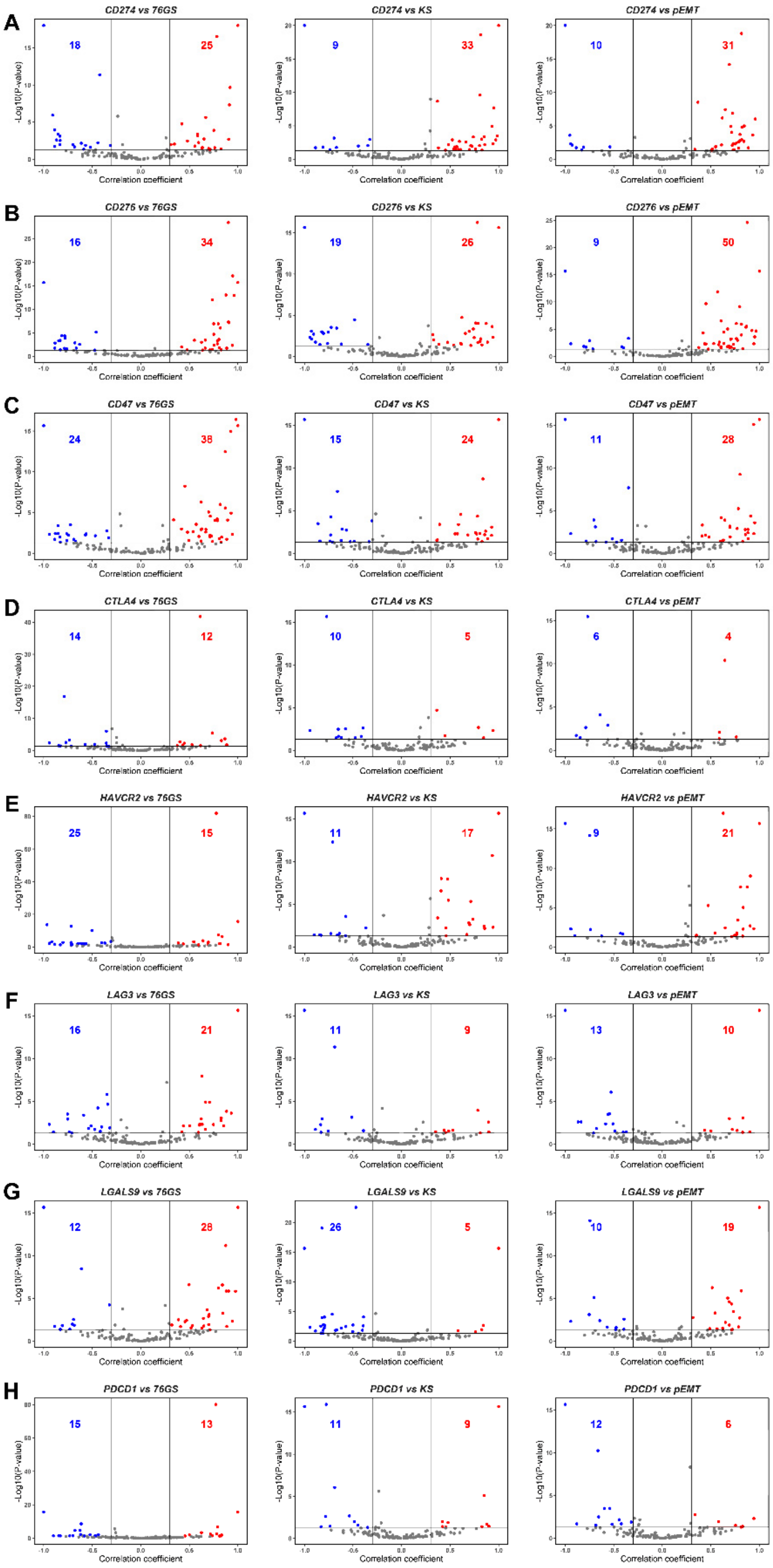

Figure S1. Relationship of immune checkpoint markers with EMT in bulk transcriptomics datasets. **A)** Volcano plots showing the correlation coefficient, 'R' (x-axis), and $-\log_{10}$(p-value) (y-axis) for CD274 gene expression vs. 76GS (left), CD274 vs. KS (middle) and CD274 vs. pEMT scores (right). Boundaries for significant correlation are set at $R > \pm 0.3$ and $p < 0.05$. Same as (**A**) but for (**B**) CD276 gene expression vs. 76GS (left), CD276 vs. KS (middle), and CD276 vs. pEMT scores (right), (**C**) CD47 gene expression vs. 76GS (left), CD47 vs. KS (middle), and CD47 vs. pEMT scores (right), (**D**) CTLA4 gene expression vs. 76GS (left), CTLA4 vs. KS (middle), and CTLA4 vs. pEMT scores (right), (**E**) HAVCR2 gene expression vs. 76GS (left), HAVCR2 vs. KS (middle) and HAVCR2 vs. pEMT scores (right), (**F**) LAG3 gene expression vs. 76GS (left), LAG3 vs. KS (middle), and LAG3 vs. pEMT scores

(right), (**G**) LGALS9 gene expression vs. 76GS (left), LGALS9 vs. KS (middle), and LGALS9 vs. pEMT scores (right), and (**H**) PDCD1 gene expression vs. 76GS (left), PDCD1 vs. KS (middle) and PDCD1 vs. pEMT scores (right).

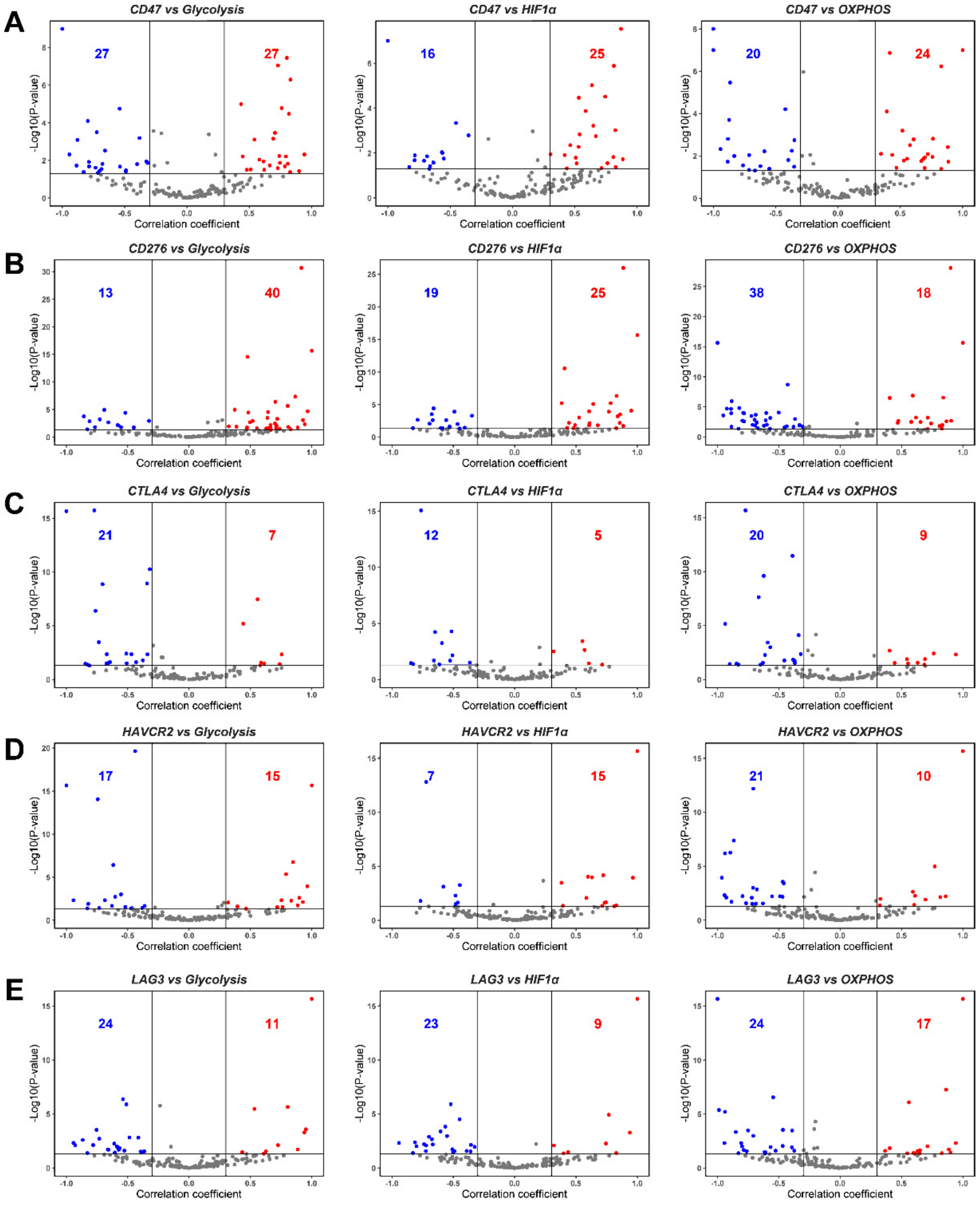

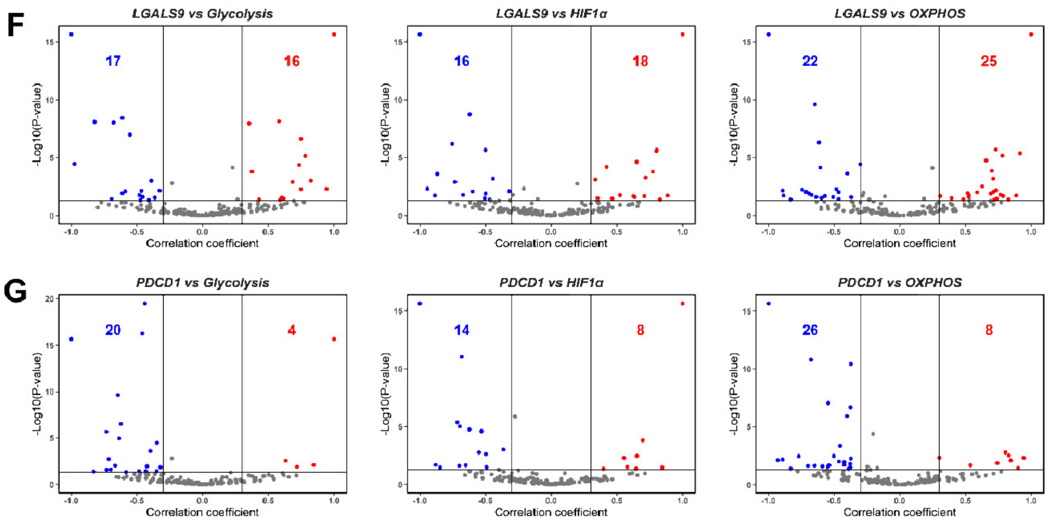

Figure S2. Relationship of immune checkpoint markers with glycolysis (its master regulator HIF-1α) and OXPHOS gene signatures. **A)** Volcano plots showing the correlation coefficient, 'R' (x-axis), and -log$_{10}$(p-value) (y-axis) for CD47 gene expression vs. glycolysis (left), CD47 vs. HIF1-α (middle) and CD47 vs. OXPHOS scores (right). Boundaries for significant correlation are set at $R > \pm 0.3$ and $p < 0.05$. Same as A) but for **B)** CD276 gene expression vs. glycolysis (left), CD276 vs. HIF1-α (middle), and CD276 vs. OXPHOS scores (right), **C)** CTLA4 gene expression vs. glycolysis (left), CTLA4 vs. HIF1-α (middle) and CTLA4 vs. OXPHOS scores (right), **D)** HAVCR2 gene expression vs. glycolysis (left), HAVCR2 vs. HIF1-α (middle) and HAVCR2 vs. OXPHOS scores (right), **E)** LAG3 gene expression vs. glycolysis (left), LAG3 vs. HIF1-α (middle) and LAG3 vs. OXPHOS scores (right), **F)** LGALS9 gene expression vs. glycolysis (left), LGALS9 vs. HIF1-α (middle) and LGALS9 vs. OXPHOS scores (right) and **G)** PDCD1 gene expression vs. glycolysis (left), PDCD1 vs. HIF1-α (middle), and PDCD1 vs. OXPHOS scores (right).

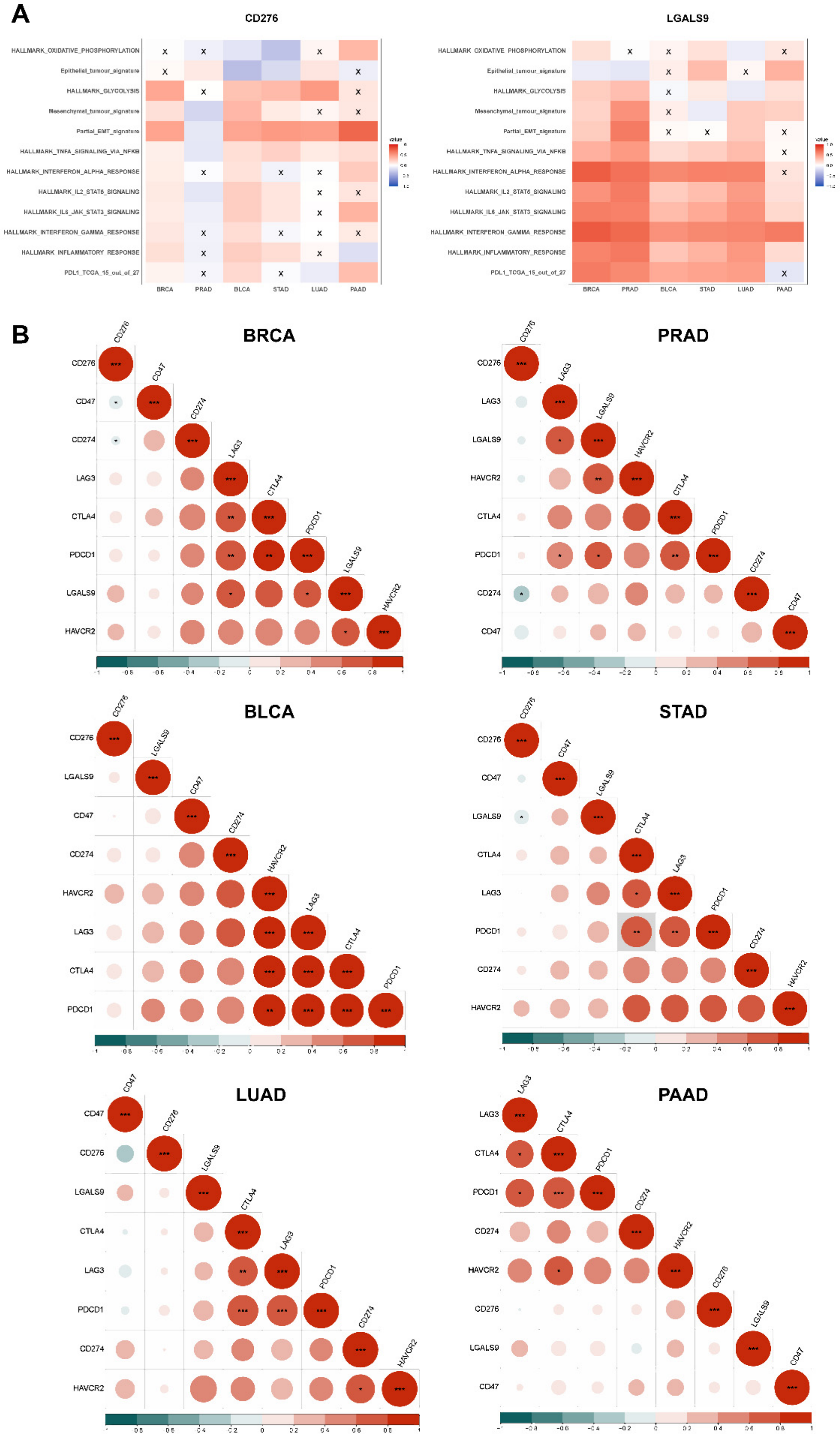

Figure S3. Association of immune checkpoint markers with hallmark pathways in TCGA datasets. **A)** Heatmap illustrating the Spearman correlation coefficients between different hallmark gene signatures with CD276 (left) and LGALS9 gene expression (right) in BRCA, PRAD, BLCA, STAD, LUAD, and PAAD. Insignificant correlations (p >0.05) are marked with 'X'. **B)** Heatmap showing correlation between different immune checkpoints in BRCA, PRAD, BLCA, STAD, LUAD, and

PAAD. p-values are calculated using unpaired Students' T-test with unequal variance and significant correlations are marked with an asterisk (*) for p < 0.05; **: p <0.01; ***: p < 0.001.

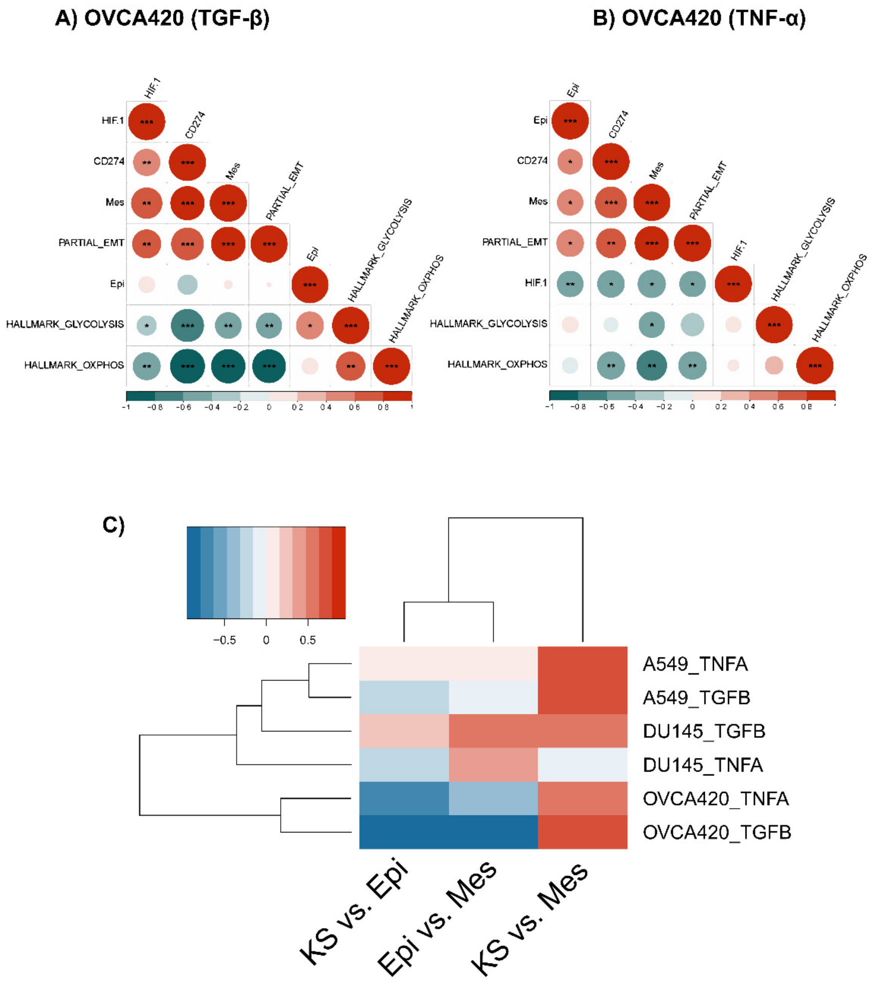

Figure S4. Association between EMT and metabolic axes with CD274 gene expression in single-cell RNA sequencing data (GSE147405). **A)** Heatmap illustrating correlation coefficient 'R' for EMT metrics, metabolic pathways, and CD274 gene in TGF-β-treated OVCA420 cell line. p-values are calculated using unpaired Students' T-test with unequal variance and significant correlations are marked with an asterisk (*) for p < 0.05; **: p <0.01; ***: p < 0.001. **B)** Same as A) but for TNF-α-treated OVCA420 cell line. **C)** Heatmap displaying Spearman's correlation coefficient for KS vs. Epi, Epi vs. Mes, and KS vs. Mes scores in A549, DU145, and OVCA420 cell lines across both treatments. All depicted correlations are significant.

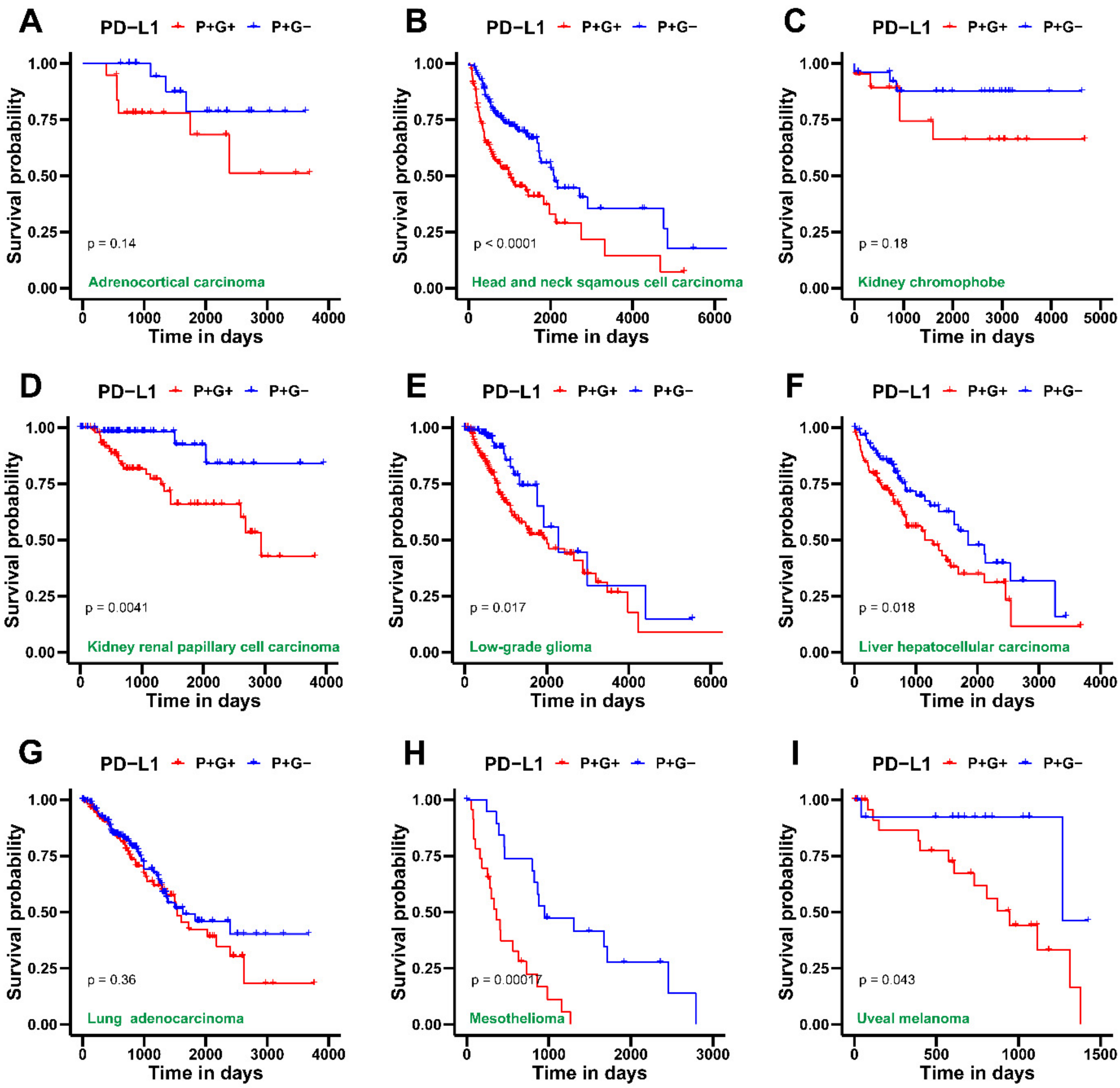

Figure S5. Survival analysis for PD-L1 and glycolysis gene signatures in several TCGA patient cohorts across cancer types. A) Kaplan–Meier curves associating overall survival (OS) with both a high PD-L1 and glycolysis signature (blue) and an enriched PD-L1 but a low glycolysis signature (red) in adenoid cystic carcinoma cohort from TCGA. Reported p-values are based on a log-rank test indicating significant difference in survival. Same as A) but for B) head and neck squamous cell carcinoma, C) kidney chromophobe, D) kidney renal papillary cell carcinoma, E) low-grade glioma, F) liver hepatocellular carcinoma, G) lung adenocarcinoma, H) mesothelioma, and I) uveal melanoma.

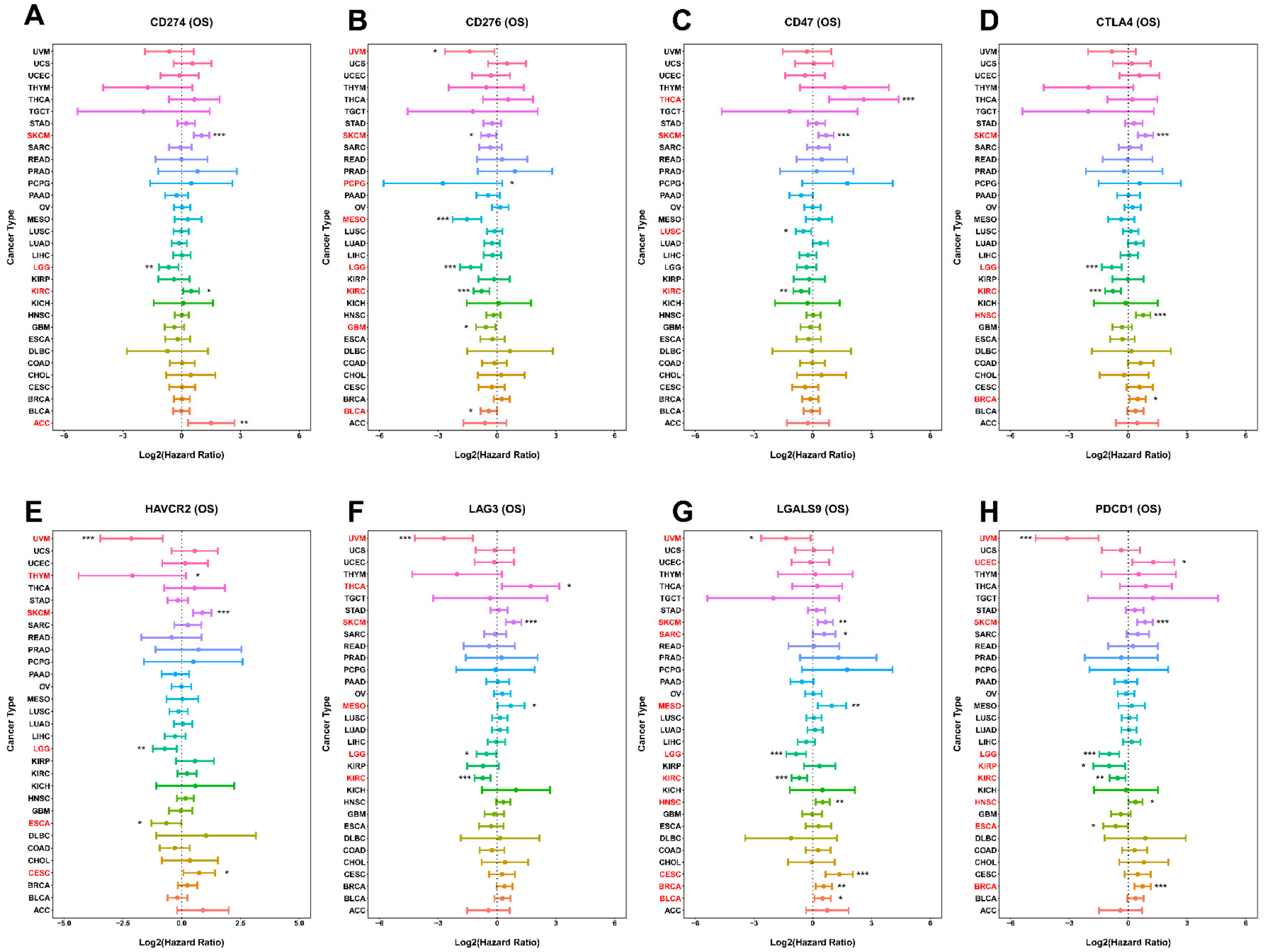

Figure S6. Cox proportional hazard ratios for immune checkpoint markers in different TCGA datasets. **A)** Forest plot depicting Log_2 hazard ratios (HR; mean ± 95% confidence interval) for overall survival associated with CD274 gene expression across several TCGA cancer types. p-values are based on log-rank test and significant difference in survival are marked with an asterisk (*) for p < 0.05; **: p <0.01; ***: p < 0.001. Axis titles of cancer types with significant differences (p < 0.05) are also highlighted in red, while insignificant ones are shown in black. Same as A) but for **B)** CD276, **C)** CD47, **D)** CTLA4, **E)** HAVCR2, **F)** LAG3, **G)** LGALS9, and **H)** PDCD1 gene expression.

Supplementary Table Legends

Supplementary Table S1A: Description of the 184 bulk transcriptomic datasets used in this study.

Supplementary Table S1B: Gene sets used for scoring EMT, metabolic and PD-L1 gene signatures.

Supplementary Table S1C: Spearman's Correlation coefficient 'R' and corresponding p-values for correlation of PD-L1 gene signature and immune checkpoint markers with KS, 76GS, Epi, Mes and pEMT scores.

Supplementary Table S2: Spearman's Correlation coefficient 'R' and corresponding p-values for correlation of PD-L1 gene signature and immune checkpoint markers with glycolysis, HIF-1α, and OXPHOS scores.

Supplementary Table S3: Spearman's Correlation coefficient 'R' and corresponding p-values for correlation of immune checkpoint markers with different hallmark pathways in TCGA cohorts.

Supplementary Table S4A: Spearman's Correlation coefficient 'R' and corresponding p-values for correlation between CD274 gene expression, E/M scores, and scores for me-tabolism in cell line samples of GSE147405.

Supplementary Table S4B: Spearman's Correlation coefficient 'R' and corresponding p-values for correlation between KS vs. Mes, Epi vs. Mes, and Ks vs. Epi for cell line sam-ples in GSE147405.

Supplementary Table S5: Log2 hazard ratios, mean ± 95% confidence intervals and corresponding p-values for overall survival associated with CD274 gene expression across several TCGA cancer types.
